# Decision-making deficits in patients diagnosed with disordered gambling using the Cambridge Gambling task: the effects of substance use disorder comorbidity

**DOI:** 10.1002/brb3.231

**Published:** 2014-04-16

**Authors:** Evangelos Zois, Noreen Kortlang, Sabine Vollstädt-Klein, Tagrid Lemenager, Martin Beutel, Karl Mann, Mira Fauth-Bühler

**Affiliations:** 1 Department of Addictive Behaviour and Addiction Medicine, Central Institute of Mental Health, Medical Faculty Mannheim, Heidelberg UniversityMannheim, Germany; 2 Kraichtal-KlinikenAm Mühlberg, 76703, Kraichtal, Germany

**Keywords:** Behavioral addiction, Cambridge gambling task, decision making, disordered gambling, substance use disorder

## Abstract

**Background:**

Disordered gambling (DG) has often been associated with impaired decision-making abilities, suggesting a dysfunction in the ventromedial prefrontal cortex (vmPFC).

**Aims:**

To our knowledge, no previous study has accurately considered the effect of substance use disorder (SUD) comorbidity (including nicotine dependence) on decision-making impairments in DG.

**Methods and Materials:**

We employed the Cambridge Gambling Task (CGT) to assess a big cohort of patients diagnosed with DG (*N = 80*) against matched healthy controls (HCs) (*N = 108*). The cohort included DG patients with nicotine and alcohol dependence, alcohol dependence only and 12 “pure” nonsmokers with only DG diagnosis.

**Results:**

*Pure* nonsmoking, nicotine dependent as well as alcoholic DGs with current nicotine dependence, demonstrated a decision making profile, characterized by poor decision-making abilities and failure to make right choices (rational), closely resembling that of patients with vmPFC damage.

**Discussion:**

This suggests that DGs with and without SUD comorbidity are equally affected in that domain of decision making abilities. Additionally, gambling diagnosis combined with alcohol and nicotine dependence involves a group of gambling patients with a relatively riskier decision making profile, showing that these patients apart from making irrational decisions take also more risks. Our findings highlight the importance of accounting for SUD comorbidities with useful implications for future research and therapy. Limitations of the current investigation are discussed.

## Introduction

Decision making is a complex cognitive process that allows people to choose the best course of action after careful consideration of the existing alternatives (Rahman et al. 2001a; Bechara 2005). Unfortunately, this ability is impaired in patients diagnosed with disordered gambling (DG) who fail to predict the negative long-term consequences of gambling (Duvarci and Varan 2000; Potenza et al. 2002). Similarly, substance-use dependent (SUD) patients seem to prefer immediate profit even in the face of negative future outcome, a finding often reporting in SUD studies using the Iowa Gambling Task (IGT) [in opioid addicts: Petry et al. (1998); alcoholics (Bowden-Jones et al. 2005) and stimulant abusers: Bechara et al. (2001); on cocaine addicts: Bolla et al. (2003)]. DG performance on the IGT is analogous to that of those with SUD (Ledgerwood et al. 2012; Leeman and Potenza 2012). Disordered gamblers (DGs) appear unable to anticipate the negative consequences associated with the risky choices they make during the task, and as a result they perform poorly (Cavedini et al. 2002; Goudriaan et al. 2005, 2006). DGs' poor decision-making abilities have also been uncovered in studies using similar task such as the game of dice (GDT) (Brand et al. 2005a,b; Labudda et al. 2007). It is suggested that impaired decision making in DGs cannot be explained by a general neuropsychological dysfunction specific to the particular patient population (Cavedini et al. 2002). It is instead a reflection of lack of insight in risky situations that irrespective of disadvantageous task performance, DGs still regard their decisions as being correct (Brevers et al. 2013).

However, neither of these tasks (IGT, GDT) differentiates between the different components of decision making, which represents a significant oversight considering that DG may leave several elements of decision making intact that might be impaired in SUD and vice versa. The Cambridge Gambling Task (CGT) is another measure of decision-making abilities with the advantage of assessing different aspects of decision-making separately, for example risky/rational choices, betting behavior, reaction time, risk adjustment (Rogers et al. 1999a,b; Deakin et al. 2004) and all that outside a learning context (Rogers et al. 1999a,b). Participants face all relevant information explicitly, allowing for the different components of decision making to be measured in standardized conditions (Rogers et al. 1999a,b; Deakin et al. 2004). To our knowledge, the only study that has so far used the CGT to investigate decision-making abilities in DG compared 21 problem gamblers to 21 alcohol-dependent subjects and 21 controls (Lawrence et al. 2009). Alcohol and gambling participants did not significantly differ in their decision-making capabilities (rational choices) compared to controls and both groups showed elevated risk taking with alcoholics being slower decision makers (Lawrence et al. 2009).

It remains unclear whether decision-making deficits in DG caused by SUD or DG itself considering that prevalence rates of SUD comorbidity in DG (Stewart and Kushner 2003) go as high as almost 60% (Black and Moyer 1998; Cunningham-Williams et al. 1998; Premper and Schulz 2008; Lorains et al. 2011), with lifetime prevalence at 73% for alcoholism and 60% for smoking (Petry et al. 2005). Existing evidence has made a distinction between those DGs with SUD comorbidity and those without suggesting that DGs with SUD constitute a group with more severe symptoms and poorer performance on measures of decision making (Petry 2001). What is more, SUD comorbidity is accountable for increased risk taking attitudes in DGs (Ledgerwood et al. 2009). While DGs with SUD have higher gambling severity index, accompanied by inferior decision-making abilities and riskier attitudes than DGs without SUD, it remains uncertain which disorder is causing which deficits in decision making. The primary aim of this study is to isolate DG from SUD and to clarify using the CGT whether gamblers' decision-making impairments are mainly caused by SUD comorbidity or by DG itself. To this end, we examined a large cohort of patients (*N* = 80) diagnosed with DG, including patients with and without SUD. We measured all of the different components of decision making as defined in the CGT (Rogers et al. 1999a,b). We expect to find impairments across all DG subgroups compared to controls with respect to rational decision making, risk taking, and response times. We anticipate finding poorer CGT performance in DGs with SUD comorbidities than those without as far as rational choices and risk taking is concerned.

## Materials and Methods

The study participants were all slot-machine-playing DG—*DG* according to the upcoming DSM-5 classification (O'Brien 2011)—patients (*N* = 80) not only recruited from the day hospital as well as inpatient treatment of the department of addictive behaviour and addiction medicine at the Central Institute of Mental Health (CIMH) in Mannheim, Germany but also from inpatient treatment centers in Münzesheim and Münchwies, both in Germany. All participants were men, met criteria for pathological gambling according to the DSM-IV (Diagnostic and statistical manual of mental disorders 2000), were between 18 and 70 years old, and were receiving treatment for gambling addiction in the form of psychotherapeutic interventions. The healthy control (HC) group (*N* = 108) was comprised of participants recruited by advertising in local newspapers as well as from a departmental recruitment pool at the CIMH. Healthy controls (HCs) were matched to DGs for age, gender, and smoking status. Participants were excluded from participation if they had a history of severe head trauma with loss of consciousness (>30 min) or if they had any neurological disease or dysfunction that might interfere with cognition. Moreover, control participants were excluded from the investigation if they had a diagnosis of any Axis I disorder according to DSM-IV [Structured Clinical Interview for DSM-IV (SCID-I)] criteria, with the exception of specific phobias. In addition, participants were excluded if they tested positive for drugs at the data collection point. SUD in both groups was assessed using SCID-I criteria for psychiatric disorders and substance dependence, DSM-IV (≥3) criteria for nicotine dependence as well as information collected from the hospitals patients were attending at the time of testing. Of the DG group, 12 had no SUD comorbidity, including no nicotine dependence (DG_pure_; we will refer to this subgroup henceforth as “pure”), 39 were nicotine dependent (DG_smoking_), 10 had a diagnosis of lifetime alcohol dependence (DG_alcohol_) and 19 meeting criteria for alcohol (lifetime) and nicotine dependence (DG_alcohol & nicotine_). In the control group, 76 subjects were nonsmokers (HC_nonsmoking_) and the remaining 32 were smokers (HC_smoking_).

**Figure 1:**
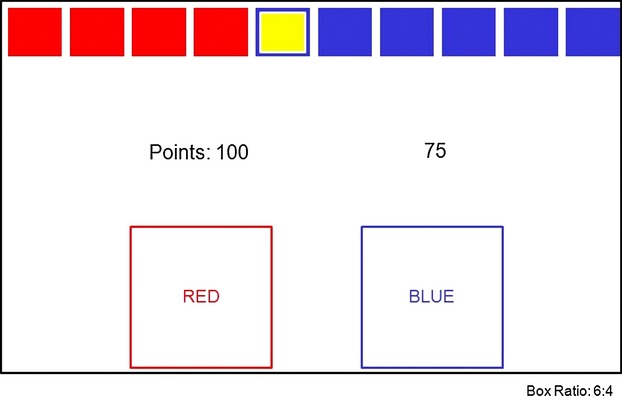
This is a schematical representation of the Cambridge gambling task. Participants viewed a computer touch screen monitor on which a total of 10 boxes (red and blue) appeared in varying ratios (6:4, 7:3, 8:2, 9:1) of red to blue. Participants had to trace a yellow token hidden inside one of these boxes. They indicated their choice by touching the appropriate box. Immediately thereafter, participants were prompted to decide on an amount to wager. If the participant had located the hidden token correctly, then the points they wagered were added to their total score. If they had made the wrong decision, however, then that same amount was subtracted from their total. Participants were always able to see their point total in the middle of the computer screen. They were able to select their bets from a list of five options calculated by the computer, with the amounts corresponding to 5%, 25%, 50%, 75% and 95% of their current point total. These bet amounts were presented either in ascending or descending order during CGT administration (ascending and descending conditions). Participants were required to choose a wager from any of these possible amounts within 2 sec. If they failed do so then the last bet was automatically set by the computer. Subjects' bets were presented together with a sound, with low-pitched tones indicating low bets, and high-pitched tones indicating high bets.

The study was approved by the medical ethics committee at the University of Heidelberg (*Ref: 2009-207N-MA*). All participants included in this investigation were taken from a large-scale study conducted in CIMH under the aegis of the Baden-Württemberg study on pathological gambling. All participants provided informed consent prior to being included in the study. We administered the South Oaks Gambling Screen (SOGS; Lesieur and Blume 1987), which is the most widely used measure of gambling severity (HCs with a score >3 were excluded) as well as the Barratt Impulsiveness Scale (BIS; Patton et al. 1995) to assess impulsivity symptoms. All subjects completed the CGT (Rogers et al. 1999a,b), a subtest of the Cambridge Neuropsychological Test Automated Battery (CANTAB, Cambridge Cognition Ltd), in order to provide information on their decision-making capabilities (Fig. 1 legend for a detailed task description). In this study, all subjects completed the ascending condition first considering that other studies have demonstrated there to be no order effect (Lawrence et al. 2009; Rubinsztein et al. 2000; Salmond et al. 2005). This study introduced a slight modification to the CGT protocol, reducing the interval between each bet to 2 sec, making the duration of the task 20 min long instead of 30 min as in the original (Nees et al. 2012), to avoid possible boredom effects. This modification did not result in “0” trials as in cases where subjects would fail to respond the computer automatically selected the last available option. All included subjects are recorded as either having won or lost points.

The different facets of decision making that are measured using the CGT include: the total number of rational choices made by the subjects, with a “rational choice” defined as that indicating the most likely outcome—highest number selection in each box ratio (quality of decision making; *QDM*); the average number of points placed on bet after the most likely outcome was chosen (risk taking; *RT*); the overall bet proportion (bet proportion; *BP*); the mean reaction time for making a selection (deliberation time; *DT*); the mean risk taking score (points) for each box ratio for both the ascending and the descending conditions where points to gamble differ relative to box ratio (risk adjustment; *RA*); and the total difference between risk-taking scores (points gambled) in the ascending and descending conditions (delay aversion; *DA*).

We used IBM SPSS V20 (Statistical Package of the Social Sciences, Version 15.0.0, SPSS Inc., Chicago, IL) to analyze the data. This being an exploratory investigation a significance threshold of *P <* 0.05 uncorrected was chosen. Data on demographic and clinical characteristics were compared using *t*-tests, one-way analysis of variance (ANOVA), and chi-squared tests. All variables from the CGT were assessed for normality (*Kolmogorov test*). Variables that did not meet the assumptions for normality were transformed using a logarithmic transformation (DT) and arcsine transformation (QDM). A 2 × 2 × 4 mixed-factor ANOVA with within-subjects factors: condition with two levels (ascending and descending), box ratio with four levels (6:4, 7:3, 8:2, 9:1) and between-subjects factor group (HCs & DGs, or HC_nonsmoking & smoking_ & DG_pure, smoking, alcohol, alcohol & nicotine_) for the outcome measures *QDM*, *RT* & *BP* was performed. Additionally, a 2 × 4 mixed-factor ANOVA was performed with box ratio four levels (6:4, 7:3, 8:2, 9:1) as within-subjects factor and between-subjects factor group (HCs & DGs, or HC_nonsmoking & smoking_ & DG_pure, smoking, alcohol, alcohol & nicotine_) for the outcome measure *RA* and finally a 2 × 2 mixed-factor ANOVA with condition as within-subjects factor (two levels) and between-subjects factor group (HCs & DGs, or HC_nonsmoking & smoking_ & DG_pure, smoking, alcohol, alcohol & nicotine_) for *DA*. Correlation analysis was performed using Pearson's method.

## Results

Demographics and clinical information are provided in detail in Table 1 for whole groups. The groups included are HCs and DGs (Table 1).

**Table 1:** Demographics and clinical characteristics (HCs vs. DGs)

	HCs	DGs	Test statistic	*P*-value
Age	36.27 (0.9)	38.13 (8.9)	1.85^F^	0.18
Marital status (single)	34%	39%	.52^*χ*2^	<0.001
Nationality (German)	97%	63%	41.03^*χ*2^	<0.001
Native speakers (German)	83%	54%	27.66^*χ*2^	<0.001
Years of education	14.53 (2.47)	11.81 (2.08)	7.51^t^	<0.001
Gambling severity (SOGS)	0.19 (0.48)	10.85 (2.89)	−37.56^t^	<0.001
Debts (%)	0.9%	82.%	133.33^*χ*2^	<0.001
DSM-IV nicotine	0.81 (0.2)	4.11 (0.3)	83.55^t^	<0.001
Impulsivity score (BIS)	59.09 (7.71)	70.28 (13.16)	−7.06	<0.001
Gambling age onset	N/A	25.13 (8.71)		

HCs, healthy controls; DGs, disordered gamblers; *t, t*-statistic; *χ*^2^, Chi-square. Values represent mean and inside the parenthesis standard deviation.

Same information is provided in Table 2 but for subgroups based on SUD comorbidities (six groups in total).

**Table 2:** Demographics and clinical characteristics (subgroups)

	HC_ns_	HC_s_	DG_pure_	DG_s_	DG_a_	DG_a&s_	*P*-value	Statistic
Age	35.1 (9.5)	39 (9.1)	38 (8.2)	35.6 (8.3)	42.3 (10.3)	41.7 (8.8)	0.018	*2.81*^*F*^
Age onset (gambling disorder)	0 (0)	0 (0)	24.2 (7.3)	23.7 (7.3)	29.3 (7.5)	26.1 (8.9)	<0.001	*233.44*^*F*^
Marital status (% single)	40%	19%	44%	39%	29%	40%	0.42	*4.94*^*χ2*^
Nationality (German)	99%	94%	42%	64%	70%	68%	<0.001	*72.11*^*χ2*^
Native speakers (German)	82%	81%	33%	54%	70%	58%	<0.001	*45.55*^*χ2*^
Years of education	14.7 (2.4)	14.2 (2.7)	10.6 (2.1)	12.1 (1.9)	11.7 (2.1)	11.8 (2.1)	<0.001	*12.03*^*F*^
BIS (impulsivity)	58.8 (8.1)	59.7 (6.7)	69.9 (13.6)	70.1 (14.5)	61.9 (8.9)	75.7 (9.2)	<0.001	*12.41*^*F*^
SOGS (gambling severity)	0.2 (0.5)	0.3 (0.5)	11 (2.8)	11.2 (2.8)	8 (2.8)	11.2 (2.9)	<0.001	*314.37*^*F*^
(%) in debt	1.3%	0	75%	80%	90%	90%	<0.001	*134.44*^*χ2*^
DSM nicotine dependence	0 (0)	2.7 (2.9)	0 (0)	5.6 (1.9)	0 (0)	5.8 (1.7)	<0.001	*98.22*^*F*^

HC_ns_, nonsmoking; HC_s_, smoking; DG_s_, smoking, DG_a_, alcohol; DG_a&s_, alcohol & smoking; *χ*^2^, Chi-square. *F*: 1 way Analysis of variance (ANOVA). Values represent mean and inside the parenthesis standard deviation. Post hoc findings in variables of interest from the above table are provided in Table 3.

**Figure 2:**
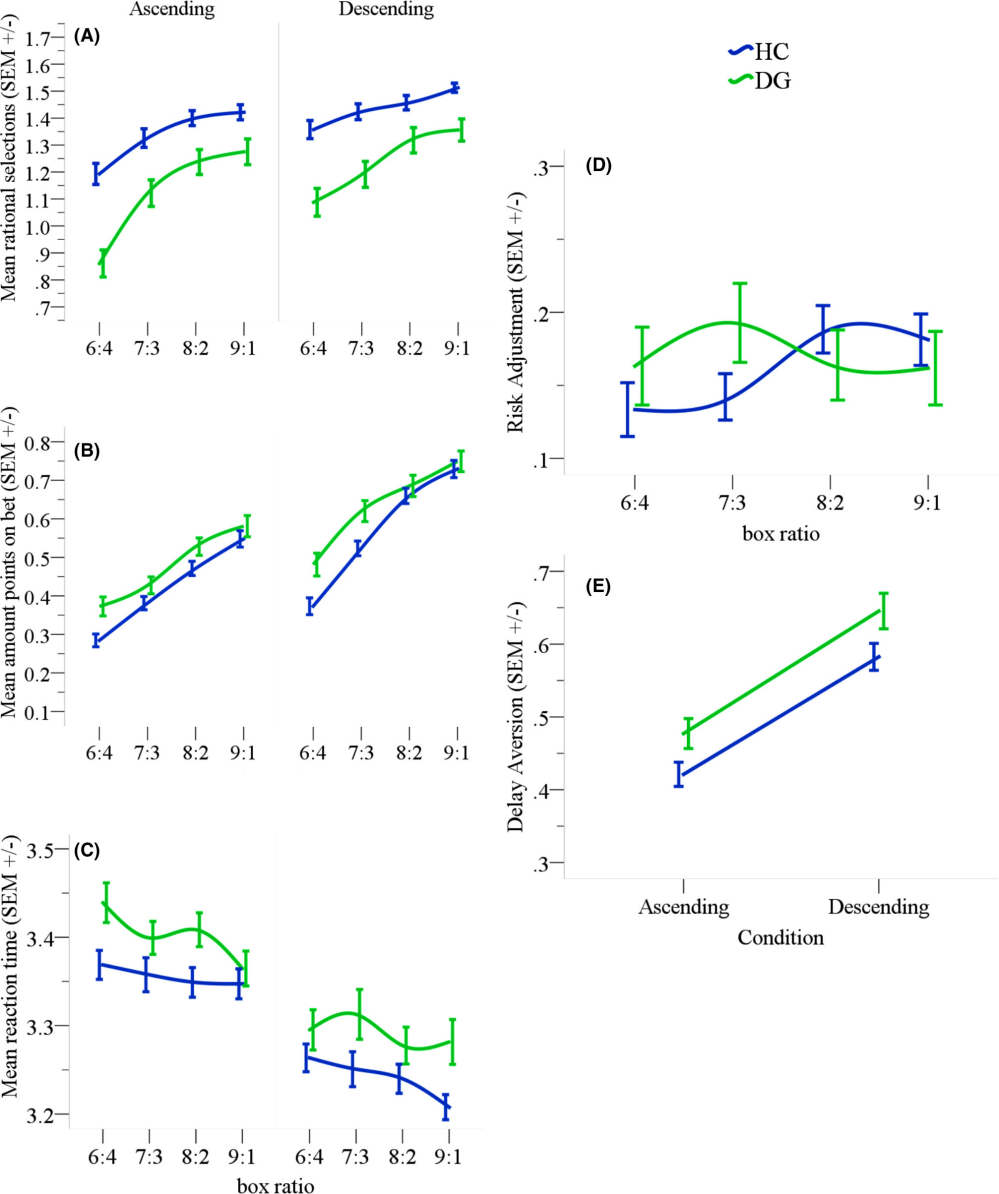
(A) Rational choices made in the task where from each box ratio the highest number chosen indicates the most likely outcome and, therefore, the right response. HCs made more rational choices than DGs. (B) Mean amount of points gambled in trials where the correct choice was made. DGs gambled more points in all box ratios compared to HCs. (C) Mean reaction times across the different box ratios for both conditions. (D) Overview of the mean number of points placed on bet across the different box ratios. HCs increased their bets relative to the increasing box ratio. DGs on the other hand placed higher bets in the early box ratios (6:4 & 7:3) and lower bets in later ratios (8:2 & 9:1). (E) Overall betting behavior for each condition separately. Both groups gambled fewer points in the ascending condition but DGs overall placed higher bets than HCs. This difference although is apparent in the graphical representation did not meet statistical significance. Error bars from the figure below represent standard error of the mean.

We also performed post hoc analysis of the variables of interest. This analysis included variables such as gambling severity and impulsivity. With regard to the CGT analysis, we have separated this into two parts too. In the first part, we conducted the statistical analysis comparing only HCs versus DGs regardless of SUD. The analysis revealed the following:

QDM: There was a significant main effect of condition and box ratio as well as a significant interaction effect between box ratio and group [*F*_(1, 186)_ = 51.6; *F*_(3, 558)_ = 68.6; *F*_(3, 558)_ = 6.2; *P <* 0.001]. There was also a significant interaction effect between condition and box [F _(3, 558)_ = 6.6, *P <* 0.001]. RT: There was a significant main effect of condition [*F*_(1, 186)_ = 150.59, *P <* 0.001], a main effect of box ratio [*F*_(3, 558)_ = 261.93, *P <* 0.001], and a significant interaction effect between box ratio and group [*F*_(3, 558)_ = 4.67, *P =* 0.003]. A significant interaction effect was also found between condition and box ratio [*F*_(3, 558)_ = 13.19, *P <* 0.001] and a significant interaction between condition, box ratio, and group [*F*_(3, 558)_ = 3.19, *P =* 0.023]. BP: There was a significant main effect of condition [*F*_(1, 186)_ = 172.98, *P <* 0.001], of box ratio [*F*_(3, 558)_ = 209.63, *P <* 0.001] and a significant interaction effect between box and group [*F*_(3, 558)_ = 5.91, *P =* 0.001]. There was also a significant two-way interaction between condition and box [*F*_(3, 558)_ = 54.75, *P <* 0.001]. DT: There was a significant main effect of condition [*F*_(1,186)_ = 145.96, *P* < 0.001] and a significant main effect of box ratio [*F*_(3, 558)_ = 8.24, *P <* 0.001]. There was also a significant interaction effect between condition, box, and group [*F*_(3, 558)_ = 3.60, *P =* 0.013). RA: There was a significant interaction effect between box ratio and group [*F*_(3, 558)_ = 3.71, *P =* 0.012]. DA: There was a significant main effect of condition [*F*_(1, 186)_ = 171.72, *P <* 0.001] (Fig. 2, whole groups).

**Figure 3:**
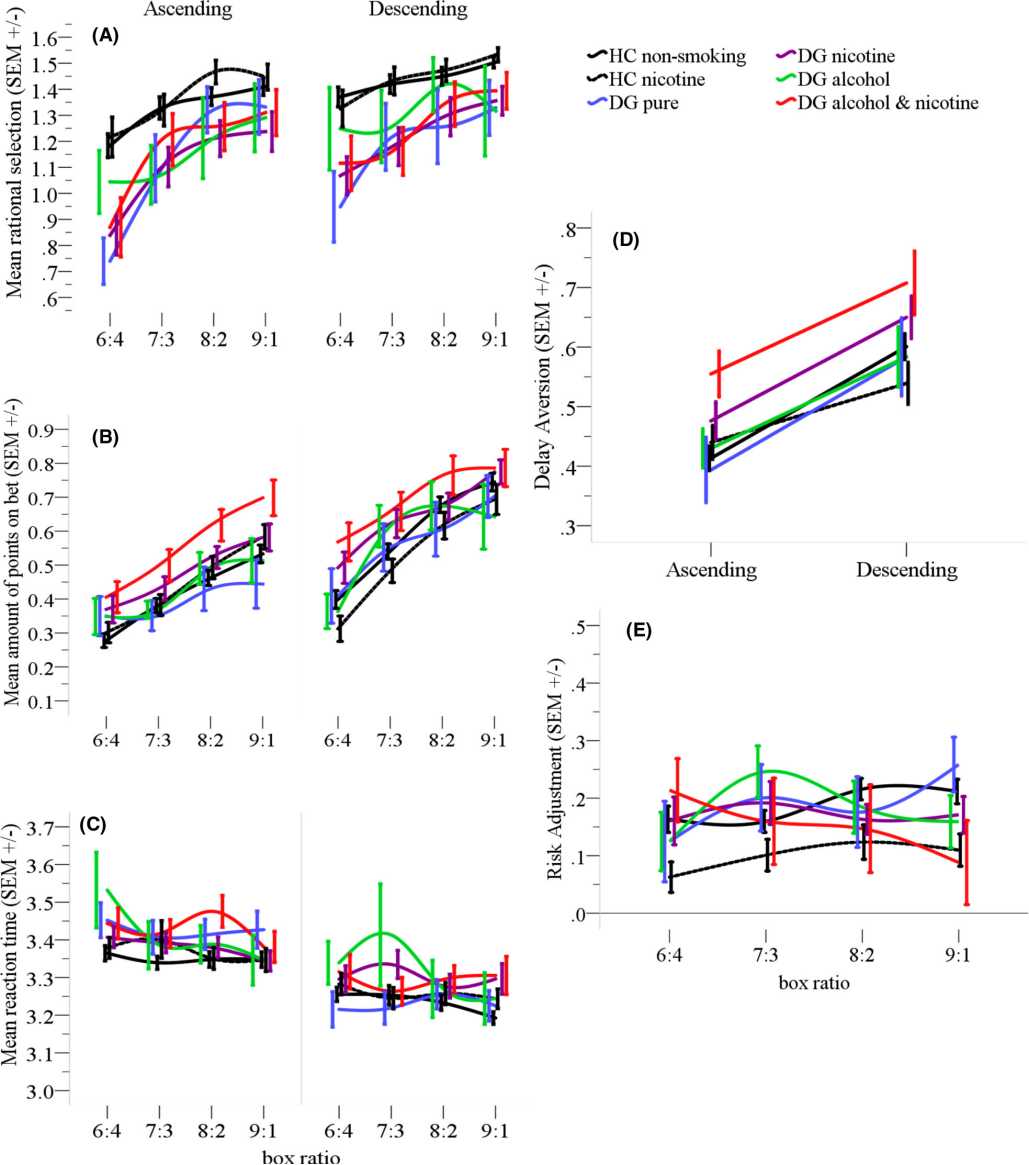
(A) Rational choices made during the task. From each box ratio the highest number indicates the most likely outcome and, therefore, the right choice. Irrational choices define the behavior in almost all DG subgroups compared to both HC groups (in the ascending phase in particular), without any difference between the DG subgroups. (B) Mean proportion of points placed on gamble across all trials regardless of whether or not the right choice was made. DG_alcohol & nicotine_ wager more than any other subgroup but only significantly different from DG_pure_ and both HC groups. (C) Mean reaction times across the different box ratios for both conditions. No significant difference was detected between any of the subgroups. (D) Overview of risk taking behavior (points gambled) across the different box ratios. Performance varies for each subgroup under investigation, however, no significant difference was detected. In this domain, subjects normally need to increase the amount they wager relative to the increasing box ratio. (E) Overview of betting behavior per condition. All subgroups bet fewer points in the ascending condition and higher in the descending. DG_alcohol & nicotine_ placed higher bets regardless of condition and were significantly different from DG_pure_ and both HC groups.

Information regarding the analysis of HCs and DGs with and without SUD (subgroup analysis) on each CGT variable separately is as follows:

QDM: There was a significant main effect of condition, a main effect of box ratio [*F*_(1, 182)_ = 30.91, *F*_(3, 546)_ = 54.23; *P <* 0.001] and an interaction effect between box ratio and group [*F*_(15, 546)_ = 2.04; *P =* 0.011]. There was also a significant interaction between condition and box ratio [*F*_(3, 546)_ = 4.83, *P =* 0.003]. DG subjects mostly chose the lowest number from each box ratio compared to both smoking and nonsmoking HCs. RT: there was a significant main effect of condition [*F*_(1,182)_ = 90.76, *P <* 0.001], a significant main effect of box ratio [*F*_(3,546)_ = 150.5, *P <* 0.001], a significant interaction effect between condition and box ratio [*F*_(3,546)_ = 12.46, *P <* 0.001] as well as a significant interaction effect between condition, box ratio, and group [*F*_(15,546)_ = 2.46, *P =* 0.002]. All groups displayed increased bets (points) relative to different box ratios but in almost all DG subgroups points put on bet were larger compared to both smoking and nonsmoking HC with more points bet in the descending condition. BP: There was a significant main effect of condition [*F*_(1, 182)_ = 104.83, *P <* 0.001]. The main effect of box ratio was also significant [*F*_(3,546)_ = 125.72, *P <* 0.001]. An interaction effect between condition and box ratio existed [*F*_(3, 546)_ = 2.63, *P =* 0.049] and there was a significant three-way interaction effect between condition, box ratio, and group [*F*_(15, 546)_ = 2.46, *P =* 0.002]. DT: A significant main effect of condition [*F*_(1, 182)_ = 112.88, *P <* 0.001] and box ratio [*F*_(3,546)_ = 7.12, *P <* 0.001] was also apparent with a significant three-way interaction effect between condition, box, and group [*F*_(12,546)_ = 2.16, *P* = 0.007]. RA: A significant interaction effect between box ratio and group [*F*_(12,546)_ = 2.11, *P =* 0.009] was found for *RA*. DA: Finally, for *DA* there was a significant main effect of condition [*F*_(1, 182)_ = 104.83, *P <* 0.001]. A detailed graphical representation of the CGT findings in relation to subgroup analysis can be seen in Figure 3.

Post hoc comparisons using the Fisher LSD test revealed significant differences between the groups (two HC and four DG groups) (see Tables 2, 3 and 4).

**Table 3:** Post hoc findings in variables of interest

Variables	Pairwise comparisons	Mean difference	*P*-value
Education (years)	HC nonsmoking > DG pure	4.01	<0.001
	HC nonsmoking > DG nicotine	2.57	<0.001
	HC nonsmoking > DG alcohol+nicotine	2.88	<0.001
	HC nonsmoking > DG alcohol	2.97	0.002
	HC smoking > DG pure	3.52	<0.001
	HC smoking > DG nicotine	2.08	<0.001
	HC smoking > DG alcohol+nicotine	2.39	<0.001
	HC smoking > DG alcohol	2.47	<0.001
BIS (impulsivity)	DG pure > HC nonsmoking	10.86	0.001
	DG pure > HC smoking	10.01	0.006
	DG nicotine > DG alcohol	9.14	0.028
	DG alcohol+nicotine > DG alcohol	14.71	0.002
SOGS (severity)	DG pure > DG alcohol	3	0.001
	DG pure > HC nonsmoking	10.8	<0.001
	DG pure > HC smoking	10.7	<0.001
	DG nicotine > HC nonsmoking	11.1	<0.001
	DG nicotine > HC smoking	10.9	<0.001
	DG nicotine > DG alcohol	3.2	<0.001
	DG alcohol > HC nonsmoking	7.8	<0.001
	DG alcohol > HC smoking	7.8	<0.001
	DG alcohol + nicotine > DG alcohol	10.9	<0.001
	DG alcohol + nicotine > HC nonsmoking	10.9	<0.001
	DG alcohol+nicotine > HC smoking	3.6	<0.001
DSM (nicotine)	HC smoking > HC nonsmoking	2.7	<0.001
	HC smoking > DG pure	2.7	<0.001
	HC smoking > DG alcohol	2.7	<0.001
	DG nicotine > HC nonsmoking	5.6	<0.001
	DG nicotine > HC smoking	2.9	<0.001
	DG nicotine > DG pure	5.6	*<0.001*
	DG nicotine > DG alcohol	5.6	*<0.001*

**Table 4:** Post-hoc findings for each CGT variable

CGT variables	Pairwise comparisons	Mean difference	*P-value*
QDM	HC nonsmoking > DG pure	0.22	0.014*
	HC nonsmoking > DG nicotine	0.21	<0.001*
	HC nonsmoking > DG alcohol+nicotine	0.17	0.018*
	HC smoking > DG pure	0.25	0.013*
	HC smoking > DG nicotine	0.21	<0.001*
	HC smoking > DG alcohol+nicotine	0.19	0.018*
RT	DG alcohol+nicotine > HC nonsmoking	0.12	0.005*
	DG alcohol+nicotine > HC smoking	0.14	0.004*
	DG alcohol+nicotine > DG pure	0.14	0.026*
BP	DG alcohol+nicotine > HC nonsmoking	0.12	0.005*
	DG alcohol+nicotine > HC smoking	0.14	0.004*
	DG alcohol+nicotine > DG pure	0.14	0.028*
DT	No significant difference		
RA	HC nonsmoking > HC smoking	0.09	0.012*
DA	DG alcohol+nicotine > HC nonsmoking	0.12	0.005*
	DG alcohol+nicotine > HC smoking	0.14	0.004*
	DG alcohol+nicotine > DG pure	0.14	0.028*

* <0.05.

Correlation analysis: QDM negatively correlated with BIS impulsivity score in DG _pure_ (*r* = −0.602, *P =* 0.043). In DG_smoking_ SOGS was positively correlated with RT (*r* = 0.39, *P =* 0.005) and DA (*r* = 0.41, *P =* 0.006). In the same group, RA was negatively correlated with BIS impulsivity scores (*r* = −0.36, *P =* 0.016). In the group DG _alcohol & nicotine_ a positive correlation was found between BIS score and RT (*r* = 0.58, *P =* 0.014) as well as DA (*r* = 0.55, *P =* 0.021).

## Discussion

The aim of this study was to explore SUD comorbidities in DG and differentiate between those decision-making impairments caused by DG and those attributable to SUD comorbidity. Initial findings (HCs vs. DGs) showed that DGs as a whole group made irrational choices in the task, accompanied by increased risk taking tendencies. Analysis in relation to SUD comorbidities revealed thereafter that DG_pure_, DG_nicotine_ and DG_alcohol & nicotine_ share a deficit in rational decision-making when compared to both nonsmoking and smoking HCs, with no significant difference between any of the DG subgroups. Elevated betting behavior mainly characterized the DG_alcohol & nicotine_ subgroup, which significantly differed from both HC groups as well as DG_pure_. In addition, DG_pure_, DG_nicotine_ and DG_alcohol & nicotine_ significantly reported higher gambling symptomatology compared to both HC groups but also DG _alcohol_. The latter did not significantly differ in impulsivity from neither HC group nor DG_pure._ Age of onset of gambling disorder for DG _alcohol_ was significantly different from that of DG_pure_ & DG _nicotine_ (later age of onset).

Decision-making deficits found are in line with earlier reports on disadvantageous choices in DGs (Grant et al. 2011; Labudda et al. 2007; Goudriaan et al. 2005; Cavedini et al. 2002). Performance in almost all DG subgroups regarding rational choices resembles that of neurological patients with damage to the vmPFC (Rogers et al. 1999b), a brain region often linked to executive function and advantageous decision making (Bechara 2005). Patients with vmPFC damage fail to make decisions to their advantage, unable to predict the long-term negative consequences of their choices (Bechara 2005). Likewise, gamblers (with and without SUD) not only chose irrationally on the CGT but also in real life by overlooking the prospective destructive effects of gambling. Performance on the particular CGT domain (QDM) seems to equally characterize addiction disorders (behavioral or not).

In another CGT domain (RT), DG_alcohol & nicotine_ were characterized by increased betting behavior and although those subjects were in remission from alcohol abuse and cognitive recovery should have occurred (Mann et al. 1999), improved executive functioning was delayed by nicotine dependence (Durazzo et al. 2007) combined with gambling diagnosis. Outside the laboratory setting these alcoholic DGs tend to engage in risky behaviors (Fernie et al. 2010), make wrong choices, reflecting the damaging effects of long-term alcohol (Bechara et al. 2001; Grant et al. 2002) joint with nicotine dependence (Durazzo et al. 2007). As a result their behavior leads to greater expenditure on gambling activities, with severer long-term consequences (i.e., higher debts). We suggest that gambling diagnosis accompanied by alcoholism and nicotine dependence represent a more challenging group of DGs (Petry 2001; Potenza et al. 2005) with implications for treatment and therapy. DG_pure_ seem to be intact when it comes to risk-taking behavior as opposed to previous findings (Lawrence et al. 2009; Grant et al. 2011). We attribute this conflicting finding to the differences in the groups assessed (treatment vs. non-treatment-seeking gamblers; alcoholics without DG diagnosis versus DGs with alcohol lifetime diagnosis). Risk taking in the form of betting might not be associated with pure behavioral addiction but rather SUD comorbidity in line with previous findings (Ledgerwood et al. 2009).

As a final remark, no cognitive abnormalities seem to accompany DG_alcohol_. They consistently made rational choices and their betting was no different from that of HCs. Possible explanation for this might relate to the fact that although protracted alcohol abuse is known to be detrimental on cognitive functioning (Harper and Matsumoto 2005), cognitive decline is reversible even within a few weeks of abstinence (Mann et al. 1999) especially in the absence of nicotine dependence. Late DG onset and the not so severe gambling index (which was also statistically verified) in the particular group suggest them to be a milder group of gamblers as opposed to gamblers with early onset of gambling disorder and severer gambling symptomatology (Burg et al. 2004; Grant et al. 2009a,b). Gambling for DG _alcohol_ appears to be at its early stages and serving as a substitute to prior alcohol dependence (Lesieur & Heinemann 1988).

Additionally, we also found associations between CGT variables and clinical variables. Irrational choices made by DG_pure_ were negatively associated with impulsivity scores; further supporting the role of impulsivity as an underlying feature of DG (Alessi and Petry 2003). Similar relationship was found in DG_smoking_ between increased impulsivity and the smaller likelihood to adjust their betting behavior to the probability of winning. In DG_alcohol & nicotine_ impulsivity scores were linked to more aggressive betting behavior, in line with previous findings (Lawrence et al. 2009; Grant et al. 2011). In almost all DG subgroups facets of decision making as measured by the CGT were mediated by impulsivity that hindered CGT performance in DGs regardless of comorbidity. Given that advantageous decision making, and inhibitory control are related to the vmPFC (Noel et al. 2006), our findings suggest an indirect association between a dysfunction of the particular brain region and addiction in general. This finding has implications for treatment outcome in DG suggesting the implementation of psychosocial therapies with a focus on impulsive behaviour (Goudriaan et al. 2008; Passetti et al. 2008). Lastly, DG_smoking_ with higher gambling symptoms tended to bet more points, supporting previous reports that nicotine dependence is related to higher gambling severity with possible adverse implications for the course of treatment and outcome in DG (Petry and Oncken 2002).

Assumptions regarding SUD comorbidity and decision making in DG can only be made for male patients. Female gamblers generally differ in terms of the onset of the disorder as well as in their attitudes toward seeking treatment (Erbas and Buchner 2012). Our findings cannot be generalized to community gamblers. As this was an exploratory study, subgroups in relation to SUD comorbidities were defined subsequent to data collection completion hence the unequal group size. Our samples also differed in terms of years of education (HCs range 9–18 years and DGs 8–17, respectively) as our HCs were not matched for years of education to our DG patients. We did not account for that difference in our analysis as years of education do not necessarily characterize cognitive abilities considering that education level per se does not influence performance on tasks measuring decision making (Bechara et al. 2000). Last but not least, the lack of premorbid IQ estimates stands as an additional limitation. The current findings are nevertheless exploratory and certainly need further validation using a study design that will not only account for differences in IQ but will also comprise of pure nonsmoking gamblers.

Gambling addiction and its neurocognitive manifestations are still relatively poorly understood. Our findings indicate that irregular decision making exists in a behavioral addiction too without being confounded by toxic effects of substances. Decision-making abnormalities seem to be a characteristic of gambling disorder itself, providing, an indirect link between decision-making deficits in “pure” behaviorally addicted individuals and dysfunction in the vmPFC brain region. DGs with comorbid alcohol and nicotine dependence seem to be more severely affected, suggesting them to be a more vulnerable group with implications for treatment course and outcome. Overall, the exploratory fashion of our results supports the recent reclassification of DG as an addiction syndrome in the new DSM-5. Nevertheless, additional research in the field is essential to construct the basis for a meaningful and unified model for addiction research and treatment. Addictions whether substance or behavioral coexist and perhaps share the same vulnerability mechanisms.
